# Identification of Pepper *AAP* Gene Family and Functional Characterization of *CaAAP6* in Drought Stress

**DOI:** 10.3390/plants15142167

**Published:** 2026-07-14

**Authors:** Jianwei Zhang, Zhinan Zhou, Xianglian Shi, Xuan Cui, Xianjun Chen, Jianwen He, Qin Yang, Huanxiu Li

**Affiliations:** 1 Guizhou Key Laboratory of Molecular Breeding for Characteristic Horticultural Crops, Kaili 556011, China; zhangjw1831@163.com (J.Z.);; 2 School of Life and Health Science, Kaili University, Kaili 556011, China; 3 Chili Pepper Research Institute, Guizhou Academy of Agricultural Sciences, Guiyang 550006, China; 4 College of Horticulture, Sichuan Agricultural University, Chengdu 611130, China

**Keywords:** pepper, AAP, subcellular localization, electron microscopy scanning, drought stress

## Abstract

Amino acid permeases (AAPs) facilitate the uptake and transmembrane transport of amino acids and play critical roles in regulating plant growth, development, and stress responses. However, the characteristics and functions of the *AAP* gene in pepper remain unclear. In this study, nine *CaAAP* genes were identified within the pepper (*Capsicum annuum*) genome and designated as *CaAAP1*-*CaAAP9*. Phylogenetic analysis revealed that *CaAAP6*, *CaAAP7*, and *CaAAP9* belong to subfamily I, *CaAAP8* is the sole member of subfamily III-E, and the remaining genes are classified within subfamily II. Collinearity analysis indicated the presence of both tandem and segmental duplication events among the nine CaAAP genes in pepper, as well as four and seven duplicated gene pairs between Arabidopsis and tomato, respectively. Expression profiling demonstrated that *CaAAP* genes exhibit pronounced tissue-specific expression patterns and differential expression in response to exogenous plant hormones and various abiotic stress treatments. Notably, drought stress elicited a significant up-regulation of *CaAAP6* expression. Functional characterization confirmed that CaAAP6 localizes to the cell membrane. Silencing of *CaAAP6* resulted in a significant reduction in plant drought tolerance, accompanied by disrupted tissue architecture, and stomatal closure. Furthermore, the concentrations of H_2_O_2_, O_2_^•−^, and MDA exhibited increases of 76.19%, 108.52%, and 20.23%, respectively. Concurrently, the activities of SOD, POD, and CAT decreased by 42.32%, 64.09%, and 109.64%, respectively. Additionally, the transcriptional levels of the *CaRD22*, *CaRD29B*, and *CaDREB2A* genes were reduced by 135.56%, 67.19%, and 37.91%, respectively, compared to the control plants. Collectively, these findings provide initial functional evidence that *CaAAP6* contributes to drought tolerance in pepper.

## 1. Introduction

The changes in rainfall patterns caused by climate change have led to a sustained high incidence of drought disasters in multiple provinces of China [1]. Drought stress has become one of the most serious abiotic stresses faced by plants in the key physiological and biochemical processes from germination to reproduction, including affecting plant water balance and disrupting photosynthesis, respiration, and amino acid metabolism. This ultimately leads to a decrease in crop yield and quality, posing a serious threat to agricultural production [2,3,4]. In order to reduce the damage caused by drought stress, plants have evolved different defense mechanisms, such as biochemical (antioxidant activity, amino acid content, phytohormones and secondary metabolites), physiological (photosynthesis, transpiration, and water transport), and morphological changes (leaf area, leaf number, and leaf senescence), to improve their survival probability [5,6].

Amino acids constitute the essential building blocks of proteins and other nitrogen-containing biomolecules, such as nucleic acids, and are integral to various physiological processes in plants. These processes include growth, development, nutrient assimilation, nitrogen fixation, energy metabolism, signal transduction, and responses to environmental stress [7]. Plants acquire nitrogen through two primary mechanisms: firstly by assimilating inorganic nitrogen sources such as nitrate (NO_3_^−^) and ammonium ions (NH_4_^+^) into amino acids, and secondly by directly absorbing amino acids from the soil [8]. Serving as the central agents for nitrogen distribution and as key constituents of plant proteins, amino acid transporters (AATs) play a vital role in plant growth and development. Based on sequence homology and transport properties, plant AATs are classified into two main families: the amino acid/auxin permease (AAAP) family and the amino acid–polyamine–choline (APC) family [9,10]. Notably, the AAAP and APC families consist of distinct sets of members.

Amino acid permeases (AAPs), belonging to the AAAP family, constitute the most extensively characterized group of amino acid transporter proteins. They play a crucial role in amino acid uptake, translocation through the xylem and phloem, regulation of seed development, and responses to environmental stress [11,12,13,14,15]. *Arabidopsis thaliana*, as a model organism, has been the subject of the most comprehensive research concerning *AAP* genes. Among these, *AtAAP1* was the first amino acid transporter identified in plants, localized to the plasma membrane of root epidermal cells, root hairs, and the entire root tip, where it facilitates amino acid uptake into root cells [16]. Subsequent research has demonstrated that *AtAAP2* is expressed predominantly in mature leaves, stems, and floral stalks, suggesting a role in the long-distance transport of amino acids [17]. *AtAAP3* exhibits root-specific expression in Arabidopsis and is implicated in amino acid absorption from the soil or phloem, while *AtAAP5* is responsible for the transport of basic amino acids such as lysine and arginine; mutants deficient in *AtAAP5* display markedly reduced amino acid uptake [18]. *AtAAP8* is critical for amino acid uptake by the endosperm, supplying essential amino acids for early embryogenesis and seed development [17]. In rice, *OsAAP5* regulates tiller formation through the transport of basic and neutral amino acids; *OsAAP6* influences amino acid distribution and starch biosynthesis; and *OsAAP17* enhances both tiller number and yield in indica rice varieties [19]. *AAP* genes in *Populus tomentosa and Nicotiana tabacum* have been associated with the transport of glutamate and glutamine [20,21]. In addition, *SiAAP3* and *-16* are implicated in the response to salt–alkali stress in foxtail millet [22]. *SlAAP6* facilitates the uptake and translocation of branched-chain amino acids, thereby enhancing tomato plant growth and conferring increased tolerance to salinity stress [15]. With respect to drought stress, the expression of tomato *SlAAP8*, *-9*, and *-10* genes is significantly up-regulated under drought conditions, concomitant with a significant reduction in NO_3_^−^ and total nitrogen (N) concentrations within both leaf and root tissues [23]. Quantitative reverse transcription polymerase chain reaction (qRT-PCR) analyses reveal that drought stress induces *ZmAAP* gene expression in *Zea mays* and *TaAAP* genes in *Triticum aestivum* [24,25]. Collectively, these studies demonstrate that *AAP* genes are integral to plant growth, development, and response to stress.

Pepper (*Capsicum annuum* L.) is a significant horticultural crop recognized for its nutritional and economic value. In China, the cultivation area of pepper exceeds 2.1 million hectares, generating an estimated total output value of approximately 250 billion yuan ($36.9 billion), underscoring its prominence in both agricultural scale and economic contribution [26]. Nevertheless, pepper production is vulnerable to various abiotic stresses, particularly drought stress, which poses a substantial threat to both the quality and yield of the crop. Previous research has indicated that *AAP* genes may play a role in the regulation of drought stress [23,24,25]. Consequently, the identification of AAP family members in pepper and the investigation of their responses to drought stress are of considerable importance. This study aims to employ bioinformatics approaches to identify and characterize the *AAP* gene family in pepper, encompassing analyses of gene structure, cis-acting elements, and phylogenetic relationships. Furthermore, the expression profiles of *AAP* genes under the influence of plant hormones and abiotic stresses will be examined, with a specific focus on elucidating the biological function of *CaAAP6* in response to drought stress. This research aims to provide significant theoretical understandings of the *CaAAP* genes and potential target genes for cultivating drought-tolerant pepper varieties.

## 2. Results

### 2.1. Identification of CaAAPs in Pepper

By aligning AAP protein sequences from Arabidopsis and rice and subsequently removing redundant and incomplete entries, nine members of the AAP family were identified within the pepper genome, designated as *CaAAP1* through *CaAAP9* (Table 1). Physicochemical analysis of these CaAAP proteins revealed sequence lengths ranging from 456 to 492 amino acids. Their molecular weights were estimated to fall between 50.68 and 55.08 kDa, while theoretical isoelectric points (pI) ranged from 8.45 to 9.10, indicating that all CaAAP proteins exhibit alkaline characteristics (pI > 7). The instability indices of these proteins ranged from 27.85 to 39.46, all below the threshold value of 40, suggesting that the CaAAP proteins are stable. Subcellular localization predictions indicated that all CaAAP proteins are localized to the plasma membrane; however, empirical validation is necessary to substantiate these predictions.

### 2.2. Analysis of Multiple Sequence Alignment and Phylogenetic Tree

To examine the similarity and conservation of AAP protein sequences, multiple sequence alignment analysis was conducted using pepper AAP protein sequences. The analysis revealed that, although all protein sequences contained the conserved transmembrane amino acid transporter domain (PF01490), the degree of sequence similarity varied among them (Figure S1). Notably, the highest similarity was observed between CaAAP2 and CaAAP5, with a similarity of 91.72%. Additionally, the sequences of CaAAP1, CaAAP2, CaAAP3, and CaAAP5 exhibited similarities exceeding 80%. In contrast, CaAAP4 demonstrated lower similarity levels with the other sequences, all below 60%, with the lowest similarity of 46.91% observed between CaAAP4 and CaAAP9.

**Figure 1:**
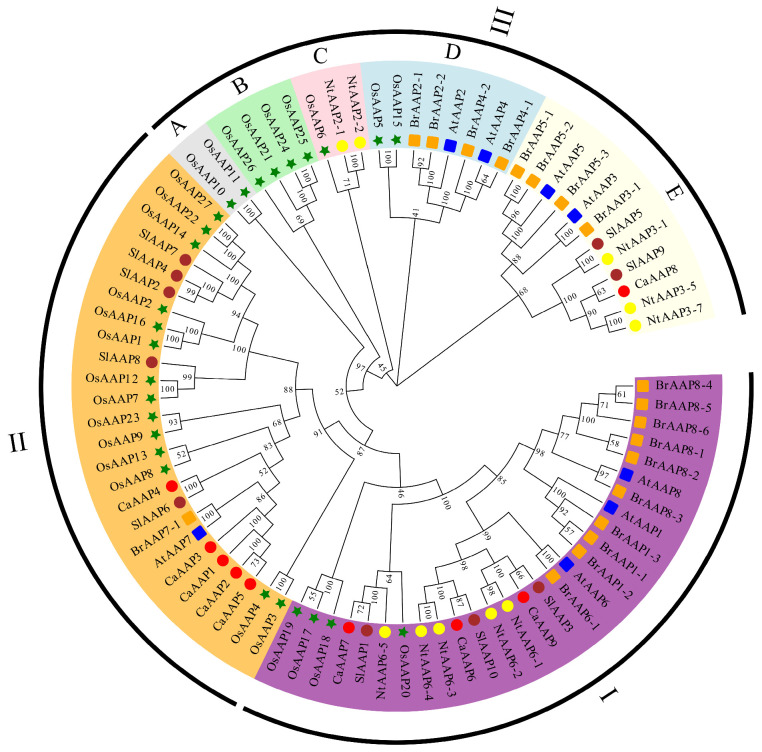
Phylogenetic tree of AAP proteins in plants. Abbreviations: Ca, *Capsicum annuum*; Sl, *Solanum lycopersicum*; At, *Arabidopsis thaliana*; Os, *Oryza sativa*; Nt, *Nicotiana tabacum*; Br, *Brassica rapa*.

A total of 83 AAP members were categorized into three primary subfamilies—I, II, and III—with subfamily III further subdivided into five distinct groups designated A through E (Figure 1). Subfamily I comprises three members (CaAAP6, -7, and -9), whereas subfamily II includes CaAAP1, -2, -3, -4, and -5. Notably, CaAAP8 is classified within group E of subfamily III. Furthermore, the analysis reveals that AAP proteins from pepper cluster closely with those from tomato and tobacco, reflecting their shared taxonomic affiliation within the *Solanaceae* family. This cluster is followed by members from the cruciferous species Arabidopsis and *Brassica rapa*, while the monocotyledonous species *Oryza sativa* exhibits a more distant evolutionary relationship with these dicotyledonous plants.

### 2.3. Preserved Motifs and Structural Characteristics of CaAAP in Pepper

**Figure 2:**
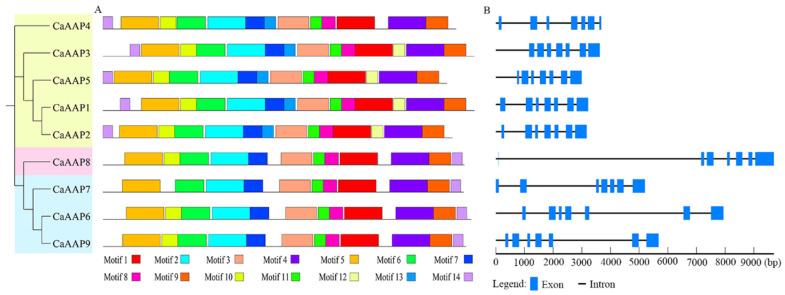
Conservative motifs and gene structures of CaAAP family members. (**A**) The distribution of conserved motifs in CaAAP proteins; conservative motifs within the *CaAAP* sequence are depicted using squares of varying colors. (**B**) Structural analysis of *CaAAP* genes.

To elucidate the genetic characteristics of the pepper amino acid permease (AAP) family, this study conducted a comprehensive analysis of the conserved motifs and gene structures of CaAAP family members. Utilizing an E-value threshold of less than 10^−3^ for motif identification, a total of 14 conserved protein motifs, designated as motif 1 through motif 14, were detected. Within subfamily II, motif 12 was exclusively found in the *CaAAP1*, *CaAAP2*, *CaAAP3*, and *CaAAP5* genes, but was absent in *CaAAP4*, reflecting the observed sequence similarity patterns. Notably, motif 14 was identified across multiple members spanning subfamilies I, II, and III; its positional distribution varied, as it was located at the N-terminus in subgroup II, whereas in subgroups I and III, it was situated at the C-terminus of the respective sequences (Figure 2A and Figure S2). Further examination of gene structures revealed that members within each subfamily exhibited comparable gene lengths, each comprising seven exons and eight introns (Figure 2B). Within subfamily II, the gene architectures of *CaAAP3* and *-5* were analogous, as were those of *CaAAP1* and *-2*. Additionally, the seventh exon of *CaAAP4* was markedly shorter relative to other subfamily members, providing a molecular basis for its reduced sequence similarity. Although *CaAAP8*, a member of subfamily III, possessed an amino acid length comparable to that of other subfamily members, it was distinguished by a notably longer intron sequence.

### 2.4. Collinearity and Selection Pressure Analysis of AAP Genes

**Figure 3:**
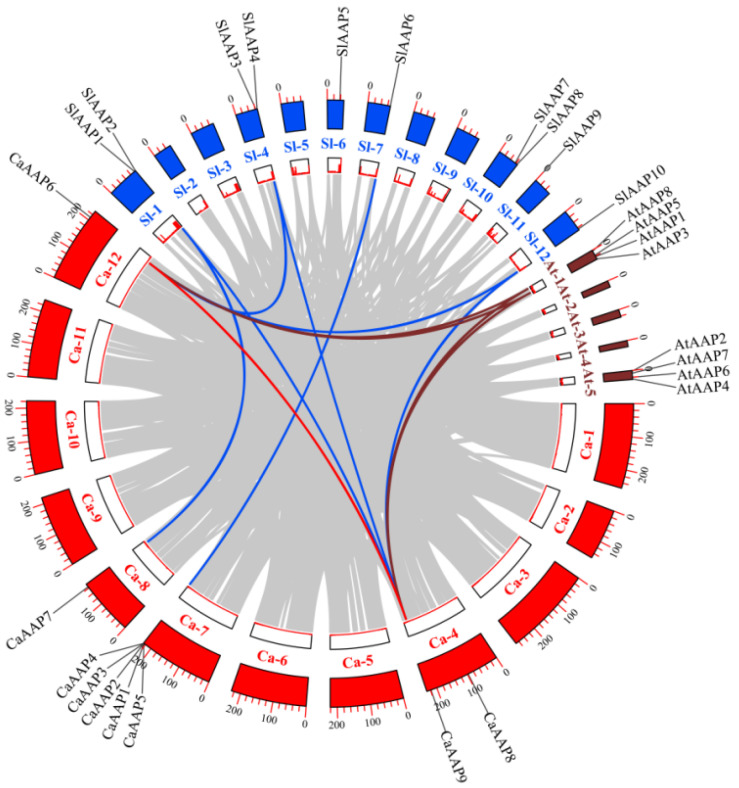
Collinearity analysis of *AAP* genes among pepper, tomato, and Arabidopsis species. Red lines denote collinear *AAP* gene pairs within the pepper genome, whereas blue and brown lines correspond to collinear gene pairs identified between pepper and tomato, and between pepper and Arabidopsis, respectively.

A collinearity analysis was conducted on pepper, tomato, and Arabidopsis AAP genes using TBtools software to investigate their evolutionary relationships across species. As shown in Figure 3, the analysis revealed that four genes within the pepper *CaAAP* gene family—*CaAAP1*, *-2*, *-3*, and *-5*—exhibited tandem duplication. This conclusion was based on their chromosomal locations being within 200 kb of each other and their protein sequence similarities exceeding 70%. Conversely, *CaAAP4* demonstrated low sequence similarities with other genes, all below 60%, indicating the absence of tandem duplication. Additionally, a pair of segmentally duplicated genes, *CaAAP6* and *CaAAP9*, was identified in pepper. Comparative analysis between pepper and tomato *AAP* genes showed a high degree of homology, with seven collinear gene pairs detected between nine *CaAAP* and ten *SlAAP* genes (blue lines). Notably, *CaAAP9* displayed collinearity with three tomato genes (*SlAAP1*, *-3*, and *-10*), while *CaAAP6* was collinear with *SiAAP3* and *-10*. Furthermore, collinear relationships were observed between *CaAAP7* and *SlAAP1*, as well as *CaAAP5* and *SlAAP6*. Between pepper and Arabidopsis, four collinear gene pairs were identified (brown lines): *CaAAP6*/*AtAAP1*, *CaAAP6*/*AtAAP8*, *CaAAP9*/*AtAAP1*, and *CaAAP9*/*AtAAP8*.

The ratio of Ka/Ks serves as a critical metric for assessing selective pressure and estimating gene duplication timing. As presented in Table 2, for the majority of AAP gene pairs (including *CaAAP6*/*CaAAP9*, *CaAAP6*/*CaAAP1*, *CaAAP9*/*CaAAP1*, *CaAAP9*/*CaAAP3*, *CaAAP9*/*CaAAP10*, *CaAAP6*/*CaAAP3*, *CaAAP6*/*CaAAP10*, *CaAAP7*/*CaAAP1*, and *CaAAP5*/*CaAAP6*), the Ks substantially exceed the Ka, resulting in Ka/Ks ratios below 1. This pattern suggests that these gene pairs are predominantly subject to strong purifying selection, with the exception of the *CaAAP9*/*AtAAP8* pair. In pepper, the estimated duplication event for *CaAAP6* and *-9* occurred approximately 47.13 Mya. In contrast, the divergence times between AAP genes in pepper and Arabidopsis range from 122.29 to 384.71 Mya. Notably, the divergence times between pepper and tomato *AAP* genes are generally more recent, spanning from 15.29 to 100.64 Mya, except for the *CaAAP5*/*SiAAP6* gene pair, which is estimated to have diverged around 243.31 Mya.

### 2.5. Analysis of Cis-Elements of CaAAP Gene Promoter in Pepper

**Figure 4:**
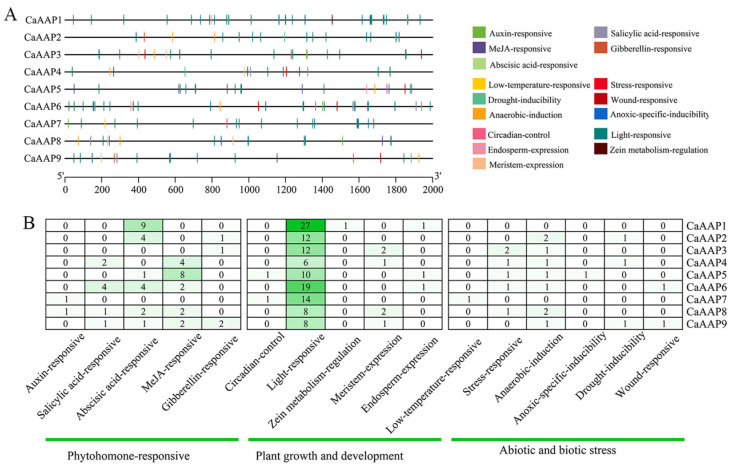
*CaAAP* family member promoter cis-acting element. (**A**) The distribution of cis-acting elements in the promoter region. (**B**) The quantity and classification statistics of cis-acting elements, the depth of color represents the number of cis-acting components.

To further elucidate the potential regulatory roles of the pepper *CaAAP* genes, a total of 203 cis-acting regulatory elements, representing 16 distinct types, were identified within the 2000 base pair promoter region upstream of the gene. These elements were categorized into three main groups: plant hormone response, growth and development, and biotic/abiotic stress response (Figure 4A). Specifically, five cis-acting elements were associated with plant growth, development, and hormone signaling, while six types pertained to biotic and abiotic stress responses.

Within the plant hormone-responsive category, ABA-responsive elements were the most abundant, totaling 21 occurrences. Notably, nine of these elements were located upstream of the *CaAAP1* gene, with four each found upstream of *CaAAP2* and *-3* genes. Additionally, 18 MeJA-responsive elements were identified, whereas auxin-responsive elements were comparatively scarce, with only two present upstream of *CaAAP2* and *-1*, respectively. Regarding elements related to biological growth and development, light-responsive elements were predominant, with most genes containing more than ten such elements. Meristematic expression elements were also detected, numbering six in total, while a single maize protein metabolism regulatory element was found exclusively upstream of the *CaAAP1* gene. In the category of biotic and abiotic stress response elements, relatively few were identified across all *CaAAP* genes. Specifically, six elements were associated with general stress induction, and nine were linked to anaerobic conditions. Three drought stress response elements were located upstream of *CaAAP2*, *-4*, and *-9* genes. Mechanical damage response elements were identified in *CaAAP6* and *-9*, whereas single elements responsive to low-temperature stress and hypoxia-specific induction were found upstream of *CaAAP6* and *-9*, respectively (Figure 4B).

### 2.6. Expression of CaAAP Genes in Different Tissues

**Figure 5:**
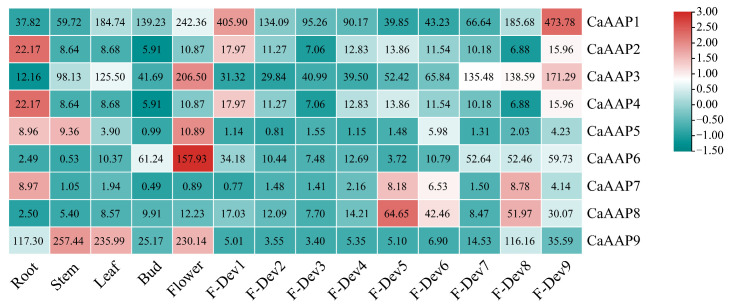
The expression profile of *CaAAP* genes in different organs and fruits at different developmental stages. F-Dev1, F-Dev2, F-Dev3, and F-Dev4 are the immature stages of the fruit; F-Dev5 is the green stage of fruit ripening; F-Dev6 is the stage of fruit color transformation; F-Dev7, F-Dev8, and F-Dev9 are the stages of fruit ripening.

Gene expression patterns across different plant tissues can provide indirect insights into their potential roles in growth and developmental processes. In this study, publicly accessible transcriptomic datasets were utilized to examine the transcriptional profiles of the pepper *CaAAP* genes in roots, stems, leaves, flower buds, flowers, and fruits at various developmental stages (Figure 5). The findings indicate that the *CaAAP1* gene exhibits elevated expression levels across multiple tissue types and throughout fruit maturation. In contrast, *CaAAP2* and *-4* show pronounced expression predominantly in roots and young fruits. Notably, *CaAAP3* and *-6* display significantly higher expression in floral organs compared to other tissues, implying a potential involvement in flower development. Additionally, *CaAAP7* and *-8* are more highly expressed during the late stages of fruit ripening, whereas *CaAAP9* demonstrates markedly increased transcription in stems, leaves, and flowers relative to other tissues.

### 2.7. Expression Patterns of CaAAP Genes Under Different Phytohormone and Stress Conditions

**Figure 6:**
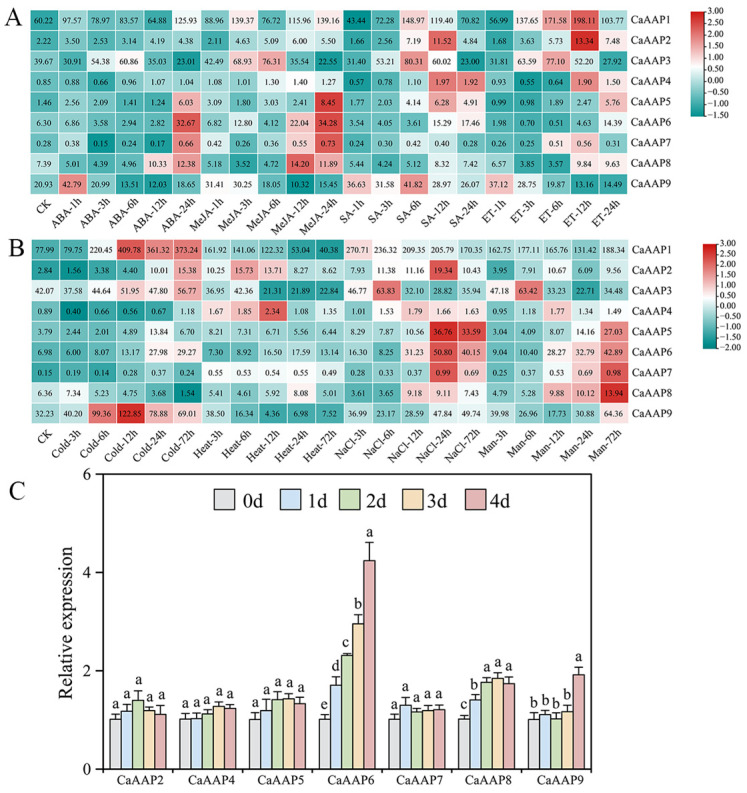
The expression levels of *CaAAP* genes under hormone and stress conditions. (**A**) The expression pattern of *CaAAP* genes under plant hormone treatment through transcriptome data analysis; (**B**) the expression pattern of *CaAAP* genes under non-biological stress treatment in plants through transcriptome data analysis; (**C**) the expression level of *CaAAP* genes under drought stress using qRT-PCR. Lowercase letters indicate statistically significant differences (*p* < 0.05).

The expression profiles of *CaAAP* genes under various plant hormone treatments were investigated utilizing transcriptomic datasets obtained from the NCBI database. The analysis revealed a significant up-regulation of these genes in response to ABA, MeJA, SA, and ET treatments (Figure 6A). Notably, the expression levels of *CaAAP1*, *-5*, *-6*, *-7*, and *-8* exhibited marked increases at the late stage (24 h) following ABA and MeJA exposure, whereas *CaAAP9* demonstrated peak expression at the early stage (1 h) of treatment. Under SA treatment, the majority of genes, including *CaAAP1*, *-2*, *-3*, *-5*, and *-9*, displayed an initial up-regulation followed by a decline. The transcriptional activity of *CaAAP6* progressively increased with extended treatment duration, while *CaAAP7* expression remained comparable to control levels. In the context of ET treatment, the temporal expression peaks of pepper *AAP* genes varied, suggesting distinct regulatory mechanisms among these genes. Regarding abiotic stress conditions, cold exposure induced elevated expression of *CaAAP1*, *-3*, and *-9*. Conversely, *CaAAP2* and *-4* were up-regulated during the early phases of heat treatment (3, 6, and 12 h) but exhibited decreased expression at later stages (24 and 72 h). Expression patterns under salt and drought stress were analogous; specifically, *CaAAP5*, *-6*, *-7*, and *-8* showed sustained transcriptional increases over prolonged treatment periods, implicating their potential roles in mediating responses to salt and drought stress (Figure 6B).

To further elucidate the role of *AAP* genes in pepper under drought stress conditions, we conducted a qRT-PCR analysis on three genes containing drought-responsive elements (*CaAAP2*, *-4*, and *-9*) as well as four putative drought-responsive genes (*CaAAP5*, *-6*, *-7*, and *-8*). The results indicated that the expression levels of *CaAAP2*, *-4*, *-5*, and *-6* initially increased and subsequently decreased during drought stress; however, these fluctuations were not statistically significant when compared to the control plants. Notably, the expression of *CaAAP6* and *-8* was significantly up-regulated in response to drought stress. Specifically, the expression of *CaAAP6* progressively increased with the length of drought exposure, reaching its maximum at 4 d of treatment, at a level 4.17 times greater than that of the control. In contrast, *CaAAP8* exhibited an initial increase followed by a decrease in expression; its expression level at 3 d was elevated by 81.58% relative to the control. Notably, no significant differences were observed in *CaAAP8* expression at 2, 3, and 4 d of drought treatment. In the case of *CaAAP9*, a significant up-regulation was observed only after four days of drought exposure, with no significant differences detected at earlier time points (1, 2, and 3 d) relative to the control. Based on these findings, subsequent investigations will focus on elucidating the molecular mechanisms underlying *CaAAP6*-mediated responses to drought stress (Figure 6C).

### 2.8. Analysis of CaAAP6 Subcellular Localization

**Figure 7:**
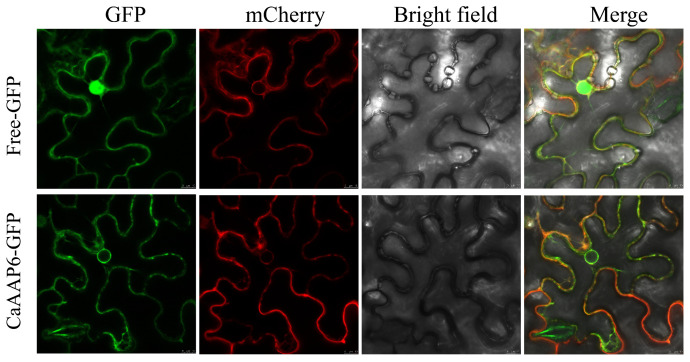
Subcellular localization of CaAAP6 in tobacco leaves. The construct PBin-AtPIP2A-mCherry serves as a plasma membrane marker, exhibiting red fluorescence. Scale bars represent 50 µm.

To investigate the subcellular localization of the CaAAP6 protein, vectors encoding Free-GFP and CaAAP6-GFP were introduced into Agrobacterium tumefaciens strain *GV3101* and subsequently co-infiltrated into tobacco leaves alongside a plasma membrane marker to facilitate fluorescence signal observation (Figure 7). The control construct, Free-GFP, exhibited fluorescence distributed across the cell membrane, cytoplasm, and nucleus. In contrast, the fluorescence signal from CaAAP6-GFP was predominantly localized to the plasma membrane of tobacco epidermal cells. These findings indicate that CaAAP6 is a protein localized to the plasma membrane.

### 2.9. Validation of CaAAP6 via VIGS for Its Potential Role in Drought Stress

**Figure 8:**
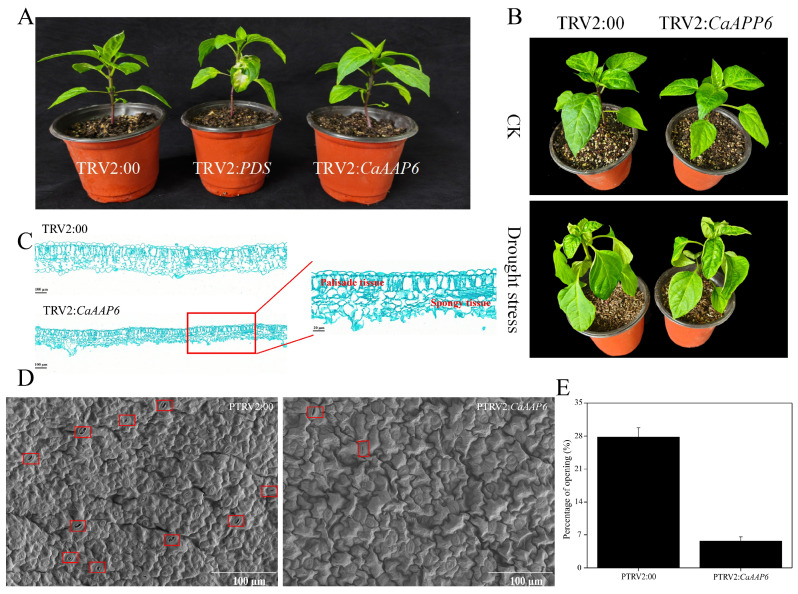
The VIGS method was used to analyze the function of *CaAAP6* in pepper under drought stress. (**A**) PDS albino phenotype, (**B**) phenotype under drought stress, (**C**) tissue staining with safranin green fixation. The image on the left was taken at a magnification of 9×, while the image on the right was taken at a magnification of 40×. (**D**) Examination of the stomata on pepper leaves using electron microscopy, with the red box highlighting stomata in an open configuration. (**E**) Quantitative assessment of the percentage of stomatal pore opening.

VIGS technology has emerged as a crucial approach for investigating gene function across various species. In the present study, silencing of the PDS gene in pepper seedlings induced a whitening phenotype, whereas no such whitening was observed in plants treated with TRV2:00 and TRV2:*CaAAP6* constructs, thereby confirming the effective silencing of the PDS gene (Figure 8A). Subsequently, qRT-PCR analysis revealed a 28–35% decrease in the expression of *CaAAP6* in silenced plants relative to TRV2:00 plants, demonstrating that these silenced plants are suitable for use in subsequent experimental procedures (Figure S3).

Under non-stress conditions, both TRV2:00 and TRV2:*CaAAP6* plants displayed normal phenotypic characteristics. Following drought treatment, leaf wilting was observed in both the control and *CaAAP6*-silenced plants; however, the degree of wilting was significantly more pronounced in TRV2:*CaAAP6* plants compared to controls (Figure 8B). Anatomical analysis using safranin green staining revealed that drought stress induced severe shrinkage of the palisade and spongy mesophyll tissues in TRV2:*CaAAP6* plants, accompanied by a reduction in intercellular spaces and a consequent increase in cell compactness. In contrast, the tissue architecture of TRV2:00 plants remained comparatively intact under the same conditions (Figure 8C). Furthermore, notable alterations in the stomatal architecture of *CaAAP6*-silenced plant leaves were identified via scanning electron microscopy (Tokyo, Japan). Under drought stress, merely 5.7% of the stomata in TRV2:*CaAAP6* plants remained open, in contrast to a stomatal opening rate of 27.8% observed in TRV2:00 plants (Figure 8D,E). These findings suggest that the silencing of *CaAAP6* exacerbates drought-induced stress and damage in pepper seedlings, necessitating a greater degree of stomatal closure to mitigate water loss from the leaves.

### 2.10. Functional Characterization of CaAAP6 in Pepper Under Drought Stress

**Figure 9:**
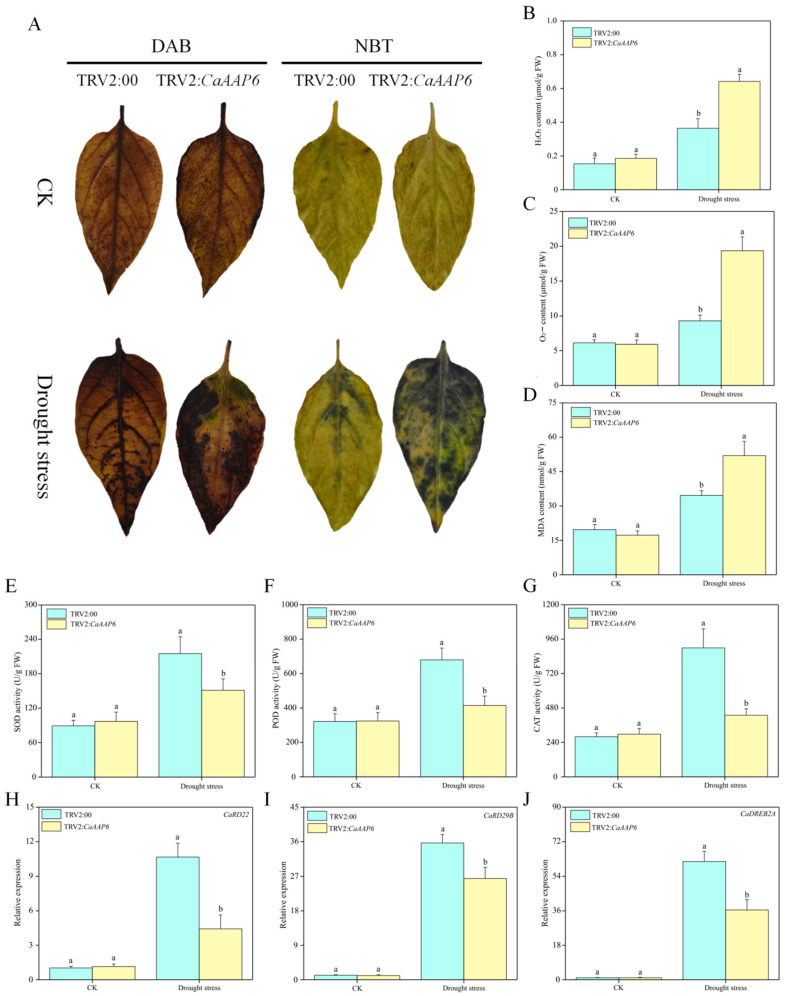
Determination of physiological and biochemical indicators alongside the expression analysis of genes in peppers under drought stress. (**A**) DAB and NBT tissue staining, (**B**) H_2_O_2_ content, (**C**) O_2_^•−^ content, (**D**) MDA content, (**E**) SOD activity, (**F**) POD activity, (**G**) CAT activity, (**H**) *CaRD22* expression, (**I**) *CaRD29B* expression, (**J**) *CaDREB2A* expression. Lowercase letters indicate statistically significant differences (*p* < 0.05).

The involvement of reactive oxygen species (ROS) in mediating physiological signaling in plants is well established. To examine the dynamic fluctuations of ROS, DAB and NBT staining assays were conducted on pepper leaf tissues. As shown in Figure 9A, both TRV2:00 and *CaAAP6*-silenced plants exhibited staining spots following drought treatment; however, the extent of staining was markedly greater in *CaAAP6*-silenced plants compared to the control. This observation aligns with the quantitative measurement data. Specifically, TRV2:*CaAAP6* plants exhibited a substantial elevation in H_2_O_2_, O_2_^•−^, and MDA concentrations under drought stress, with increases of 76.19%, 108.52%, and 20.23%, respectively, relative to the control group (Figure 9B–D). Furthermore, the antioxidant enzyme activities of SOD, POD, and CAT were assessed (Figure 9E–G). Under non-stress conditions, no significant differences were detected between TRV2:*CaAAP6* and TRV2:00 plants. For drought treatment, the activities of these enzymes in *CaAAP6*-silenced plants decreased by 42.32%, 64.09%, and 109.64%, respectively, compared to the control plants. Subsequently, the expression levels of drought-responsive genes were examined by qRT-PCR; the silencing of *CaAAP6* resulted in a significant reduction in the expression of *CaRD22*, *CaRD29B*, and *CaDREB2A* genes (Figure 9H–J). Collectively, these findings indicate that silencing the *CaAAP6* gene diminishes the antioxidant capacity, attenuates the expression of drought-responsive genes, and consequently impairs the drought tolerance of pepper seedlings.

## 3. Discussion

The *AAP* gene family is integral not only to the uptake, transport, and assimilation of amino acids but also to a range of physiological processes including plant growth, organ development, and responses to abiotic stressors. To date, members of the *AAP* family have been characterized in several species, such as *Arabidopsis thaliana* [16,27], *Oryza sativa* [13,19], *Solanum lycopersicum* [23], *Nicotiana tabacum* [21], *Solanum tuberosum* [28], *Cucumis sativus* [29], *Camellia sinensis* [30], and *Brassica napus* [31]. However, investigations into the *AAP* gene family in pepper remain unexplored. In this study, we identified nine genes belonging to the *AAP* family in pepper. The observed minor variations in amino acid length and molecular weight of the encoded proteins were consistent with findings reported in previous studies on tomato and wheat [21,32]. Furthermore, multiple sequence alignment of CaAAP proteins alongside conserved domains characteristic of transmembrane amino acid transporters revealed a high degree of sequence conservation within the AAP protein family.

A phylogenetic tree represents the evolutionary relationships among species or gene families and serves as a fundamental tool in contemporary biological research [33]. In this study, the quantity of *CaAAP* genes exhibits a strong correlation with that of *SlAAP* genes (Figure 1). Furthermore, members of the pepper *CaAAP* family within the same subfamily display conserved gene structures characterized by identical exon and intron counts, indicating a high degree of conservation of the *CaAAP* gene. Tandem repeat sequences contribute to the formation of gene clusters, whereas segmental duplications give rise to homologous genes; both mechanisms are implicated in the expansion of gene families [34]. In the present study, five genes were found in close proximity on chromosome 7, with *CaAAP1*, *-2*, *-3*, and *-5* constituting a tandem repeat gene cluster. This genomic arrangement suggests potential co-expression and involvement in the regulation of specific biological processes. Similar tandem repeat patterns have been observed in rice and maize [19,24]. Segmental duplications were also detected within the *CaAAP* gene family, exemplified by *CaAAP6* and *-9*, indicating that both tandem and segmental duplications have played significant roles in the evolutionary trajectory of the pepper *AAP* gene family. The Ka/Ks is commonly employed to infer selective pressures acting on gene duplicates [35]. In this study, the estimated divergence time of AAP sequences between pepper and Arabidopsis substantially exceeds that between pepper and tomato, corroborating previous findings regarding species divergence timelines [36,37].

Cis-acting elements located within promoter regions are integral to the regulation of gene expression [38]. This study identified several cis-acting elements within the *CaAAP* gene family, indicating that CaAAP protein genes may play a role in plant growth, development, and stress response mechanisms. Previous research has demonstrated tissue-specific expression patterns of *AAP* genes in plants. For instance, *AtAAP3* transcription in Arabidopsis predominantly occurs in roots, whereas *-4* and *-5* are primarily expressed in mature leaves rather than young leaves [39]. In rice, *OsAAP7* and *-9* exhibit significantly elevated expression during vegetative, reproductive, and leaf maturation stages, including in leaf sheaths and roots, while *OsAAP15* is notably up-regulated in anthers, embryos, and embryonic disks [19]. Similarly, in tea plants, *CsAAP14* shows relatively high expression in leaves, whereas *CsAAP19* expression is restricted to roots [29]. Within this study, *CaAAP3* and *-6* displayed high expression levels in flowers, whereas *CaAAP2* and *-4* were predominantly expressed in roots. The gene expression profiles observed in various tissues underscore their critical involvement in mediating responses to abiotic stress [40]. For example, the expression of several *CaAAP* genes in pepper, including *CaAAP1*, *-5*, *-6*, and *-7*, was modulated by plant hormones and stress conditions. Previous studies have shown that tomato genes *SlAAP8*, *-9*, and *-10* are up-regulated under drought stress [23], and that certain maize *AAP* genes are similarly induced by drought [24]. Interestingly, our analysis revealed that, under drought stress, the expression levels of the *CaAAP2*, *-4*, and *-9* genes—each containing drought-responsive elements within their promoter regions—did not exhibit significant differences relative to the control plants. Conversely, *CaAAP6* demonstrated up-regulated expression in response to drought stress, despite lacking drought-responsive elements in its promoter region. This finding implies that *CaAAP6* may be involved in the drought stress response via indirect mechanisms or through interactions with alternative signaling pathways.

Drought stress, recognized as a critical abiotic stress, exerts a profound adverse effect on plant growth and development, as well as crop quality and yield [41]. Prior investigations have demonstrated that the expression of *AAP* genes is responsive to drought conditions [21,24]. In the present study, VIGS was utilized to further elucidate the functional role of the drought-responsive gene *CaAAP6*. Compared to wild-type plants, those subjected to *CaAAP6* silencing exhibited a marked reduction in drought tolerance, which correlated with structural damage observed in the palisade and spongy mesophyll tissues attributable to dehydration (Figure 8B,C). H_2_O_2_ and O_2_^•−^ are inevitable byproducts of aerobic metabolism; under drought stress, elevated ROS accumulation can precipitate oxidative stress and consequent tissue injury in plants [42]. In this study, *CaAAP6*-silenced plants subjected to drought stress exhibited significantly higher accumulations of H_2_O_2_ and O_2_^•−^ compared to control plants, concomitant with reduced activities of antioxidant enzymes and expression levels of drought-responsive genes, implying that *CaAAP6* may enhance pepper drought tolerance through the modulation of antioxidant enzyme activity and improvement of intracellular ROS scavenging efficiency (Figure 9E–J). Nonetheless, the precise regulatory mechanism by which *CaAAP6* responds to drought stress in pepper, including whether this response operates via ABA-dependent or ABA-independent pathways, remains to be elucidated through further investigation.

## 4. Materials and Methods

### 4.1. Pepper Materials and Drought Stress

The experimental material utilized in this study was the ‘Chuannong pickled pepper.’ Seeds exhibiting plumpness were selected and soaked in water to facilitate germination. Subsequently, the seeds were sown in a tray containing seedling substrate with 60 holes. Upon reaching the developmental stage characterized by four true leaves and one central leaf in the seedling chamber, the seedlings were transferred to nutrient bowls for continued growth. The cultivation parameters were regulated under a photoperiod of 16 h of light followed by 8 h of darkness, with a light intensity set at 6500 lux. Temperature conditions were cycled between 23 °C and 27 °C, while relative humidity was maintained at 70%. When the seedlings developed eight true leaves and one central leaf, they were subjected to drought stress treatment. The experiment was conducted to simulate drought conditions by withholding irrigation. At 18:00, 50 mL of water was administered to each nutrient container, with the following day defined as the initial day (0 d) of drought stress. Thereafter, leaf samples were collected at 0, 1, 2, 3, and 4 d following the initiation of drought treatment, rapidly frozen in liquid nitrogen, and subsequently stored at −80 °C for further analysis.

### 4.2. Identification and Physicochemical Analysis of CaAAP Genes in Pepper

The genome protein sequence and corresponding annotation file for the pepper cultivar ‘UCD-10X-F1’ were obtained from the NCBI database (https://www.ncbi.nlm.nih.gov/, accessed on 22 October 2025). Annotation data and protein sequences for the Arabidopsis *AAP* gene were retrieved from the Arabidopsis Information Resource (TAIR) database (https://www.arabidopsis.org/, accessed on 22 October 2025). Additionally, pertinent datasets for rice, tobacco, tomato, and cabbage were acquired from the Ensembl Plants database (https://plants.ensembl.org/index.html, accessed on 23 October 2025).

To identify members of the pepper AAP family, a BLAST search was conducted using eight AAP protein sequences from Arabidopsis and twenty-seven sequences from maize, applying a significance threshold of 1 × 10^−10^ [43]. Concurrently, Pfam and CD-search tools were employed to detect protein sequences containing the conserved transmembrane amino acid transporter domain (PF01490). Redundant or incomplete domain sequences were subsequently excluded, resulting in the final set of sequences classified as members of the pepper AAP family. Furthermore, the physicochemical properties of the CaAAP protein sequences in pepper were analyzed via the ExPASy platform (http://web.expasy.org/protparam/, accessed on 5 November 2025), and their subcellular localization was predicted using the CELLO web server (https://cello.life.nctu.edu.tw/, accessed on 5 November 2025).

### 4.3. Evolutionary Tree Construction and Multiple Sequence Alignment

Protein sequences of the AAP family from various species—including Arabidopsis (8 members), rice (27 members), tomato (10 members), tobacco (10 members), cabbage (19 members), and pepper (9 members)—were analyzed using MEGAX64 software (version 10.0.5) to construct a phylogenetic tree via the maximum likelihood method. The bootstrap value was set to 1000, and the JTT model was employed, while all other parameters remained at their default settings. The resulting phylogenetic tree was subsequently refined and visualized using the EvolView online platform (http://www.evolgenius.info/evolview/, accessed on 27 November 2025). Additionally, multiple sequence alignment of the structural domains in pepper was conducted using ClustalX software (version 2.1), and sequence similarity was assessed through BLASTP searches within the NCBI database.

### 4.4. Select Pressure and Collinearity Analysis

Tandem repeat genes are defined as two adjacent genes located on the same chromosome, separated by less than 100 kilobases, and exhibiting a sequence similarity exceeding 70% [44]. Fragmental duplication events among members of the *AAP* gene family were analyzed using the MCScanX [45]. To assess selective pressure, the nonsynonymous substitution rate (Ka), synonymous substitution rate (Ks), and their ratio (Ka/Ks) were calculated employing DnaSP software (version 6) [46]. The evolutionary duplication time (T) was estimated using the formula T = (Ks × 10^−6^)/(2λ), expressed in million years ago (Mya), where the substitution rate constant (λ) for pepper is 7.85 × 10^−9^ [36].

### 4.5. Conserved Motifs and Gene Structure Analysis of Members of the Pepper CaAAP Family

The MEME tool (https://meme-suite.org/, accessed on 12 December 2025) was employed to analyze conserved motifs within CaAAP proteins. Utilizing a screening criterion of E-value < 0.01, the number of conserved motifs was set to 14. Furthermore, genomic annotation data were used to retrieve the positional information of exons and introns of the pepper *CaAAP* genes on the chromosomes, facilitating the construction of gene structure maps.

### 4.6. Analysis of Cis-Acting Elements in Pepper CaAAP Gene Promoter

The 2000 bp upstream sequence of the *CaAAP* gene promoter was retrieved from the pepper genome DNA sequence. Subsequently, this sequence was utilized on the PlantCARE web platform (https://bioinformatics.psb.ugent.be/webtools/plantcare/html/, accessed on 12 December 2025) to predict cis-acting regulatory elements.

### 4.7. Expression Analysis of CaAAP Members in Pepper

To examine the expression profiles of *CaAAP* genes across various tissues of pepper, transcriptomic data (PRJNA193077) corresponding to the ‘Zunla No.1’ cultivar at multiple developmental stages were retrieved from the NCBI database [37]. These data encompassed samples from roots, stems, leaves, flower buds, flowers, green ripe fruits (F-Dev1, F-Dev2, F-Dev3, and F-Dev4), color-changing fruits (F-Dev5), and red ripe fruits (F-Dev6, F-Dev7, F-Dev8, and F-Dev9).

The expression patterns of the pepper *CaAAP* genes were analyzed under plant hormone treatments (PRJNA634831) and abiotic stress conditions (PRJNA525913) utilizing publicly accessible transcriptomic datasets [47,48]. The hormone treatments encompassed abscisic acid (ABA), methyl jasmonate (MeJA), ethylene (ET), and salicylic acid (SA). Samples were collected at 1, 3, 6, 12, and 24 h post-treatment, with untreated pepper seedlings serving as controls. Abiotic stress treatments included exposure to cold, heat, drought, and salinity. Sampling occurred at 3, 6, 12, 24, and 72 h following treatment, with untreated pepper plants as the control group. The Hisat2 (version 2.2.1), Stringtie (version 2.1.7), and ballgown (version 4.4.0) software packages were used to process RNA-seq data. Gene expression levels were quantified using FPKM (Fragments Per Kilobase of transcript per Million mapped reads) values, and heatmaps were generated employing TBtools software (version 2.467) to visualize expression profiles [49].

### 4.8. RNA Extraction and qRT-PCR

Total RNA was extracted from pepper leaf samples utilizing the FastPure Plant Total RNA Isolation Kit (Novozymes, Nanjing, China). RNA concentration and purity were assessed with an ultra-micro nucleic acid protein analyzer (Ximei, Shanghai, China), ensuring that the OD260/280 ratio ranged between 1.8 and 2.1. Complementary DNA (cDNA) synthesis was performed using the Hiscript III RT Super Mix kit (Novozymes, Nanjing), following the manufacturer’s protocol. Gene-specific primers were designed via the Primer3 online tool, and their specificity was validated using the Primer-BLAST function available through the NCBI database. Primer sequences are listed in Table S1. The CaUbi3 gene (*LOC107873556*) was employed as the internal reference for normalization [50].

qRT-PCR analysis was conducted utilizing the 2X SYBR Green Fast qPCR Mix (Novozymes, Beijing, China) on a Bio-Rad CFX96 PCR system (Bio-Rad, Hercules, CA, USA). The reaction mixture comprised 10 µL of 2X SYBR Green Fast qPCR Mix, 0.5 µL of forward primer (10 µM), 0.5 µL of reverse primer (10 µM), 1 µL of cDNA template, and 3 µL of double-distilled water (ddH_2_O). The thermal cycling protocol included an initial denaturation step at 95 °C for 30 s, followed by 39 cycles of denaturation at 95 °C for 10 s and annealing at 55 °C for 30 s. Subsequently, a melting curve analysis was performed, ranging from 65 °C to 95 °C with increments of 0.5 °C every 5 s. Relative gene expression levels were quantified using the 2^−ΔΔCt^ method [51]; each gene expression measurement includes three biological replicates and three technical replicates.

### 4.9. Subcellular Localization of CaAAP6 in Pepper

The *CaAAP6* gene was amplified using specific primers, followed by detection and purification via agarose gel electrophoresis. Subsequently, the purified fragment was ligated into the subcellular localization vector pBI121-GFP to generate the fusion expression construct CaAAP6-GFP, which was then subjected to sequencing for verification. Both the fusion construct and an empty vector control were transformed into four-week-old Nicotiana benthamiana plants. The transformed plants were initially incubated in darkness for 24 h, followed by exposure to low-light conditions for 12 h. Fluorescence signals were observed using a Leica TCS SP8 laser confocal microscope to determine the subcellular localization of CaAAP6. Image analysis was performed utilizing the Leica Application Suite X software.

### 4.10. VIGS-Mediated Silencing of CaAAP6 Gene

A 280 bp fragment of *CaAAP6* was selected for gene silencing utilizing the SGN VIGS platform (https://vigs.solgenomics.net/, accessed on 15 March 2026) [52]. The Primer BLAST tool was employed to assess the specificity of the silencing fragment, thereby enhancing the efficiency of gene silencing. The recombinant vector PTRV2:*CaAAP6* was constructed via double digestion with the restriction enzymes XbaI and BamHI. Following verification, the recombinant plasmid was introduced into Agrobacterium tumefaciens strain GV3101. Cultures of PTRV1, PTRV2:00, PTRV2:*CaAAP6*, and PTRV2:*PDS* were separately grown with shaking until reaching an optical density at OD_600_ of 0.8 to 1.0. Bacterial cells were then harvested by centrifugation and resuspended in a solution containing 1 M MgCl_2_, 1 M MES, and 50 mM acetosyringone (AS). The suspension was adjusted to an OD_600_ of 0.6. Equal volumes of TRV2:00, TRV2:*PDS*, or PTRV2:*CaAAP6* suspensions were mixed with TRV1 in a 1:1 ratio, followed by incubation with shaking at 50 rpm in the dark for 2 h. The resulting mixtures were infiltrated into the cotyledons of two-week-old pepper plants. After 48 h of incubation in darkness, the plants were transferred to standard growth conditions for continued development. A total of 50 control plants and gene-silenced plants were utilized in the study. Twenty-one days post-inoculation, the silencing efficiency of the *CaAAP6* gene was assessed in gene-silenced plants. When seedlings reached the stage of eight true leaves and one heart leaf, they were subjected to drought stress treatment for 4 d, during which irrigation was withheld to simulate drought conditions. Leaf samples were subsequently collected and stored at −80 °C in liquid nitrogen for further analysis.

### 4.11. Determination of Physiological Indicators

The physiological and biochemical parameters assessed in this study encompassed hydrogen peroxide (H_2_O_2,_ AKAO009M), superoxide anion (O_2_^•−^, AKAO008M), and malondialdehyde (MDA, AKFA013M) content, alongside the enzymatic activities of superoxide dismutase (SOD, AKAO001M), peroxidase (POD, AKAO005M), and catalase (CAT, AKAO003-2M). Kits procured from Beijing Bosi Biotechnology Co., Ltd., (Beijing, China) were utilized for these assays. The procedures were conducted in accordance with the manufacturer’s protocol, and the content or enzymatic activity was determined relative to the mass of the tissue sample.

To facilitate the chemical detection of H_2_O_2_ and O_2_^•−^ in pepper leaves, staining techniques employing 3,3′-diaminobenzidine (DAB) and nitroblue tetrazolium (NBT) were utilized. The working solutions were prepared at concentrations of 1 mg/mL for DAB and 0.5 mg/mL for NBT, respectively. Fresh leaves were washed and dried before being placed in a plant tissue culture bottle. Subsequently, 150 mL of the staining solution was added, and the samples were incubated in the dark for 24 h. Following incubation, the leaves were boiled in 95% ethanol to decolorize, after which photographic documentation was performed for further analysis [53]. Furthermore, safranin green staining and scanning electron microscopy analyses were performed by Wuhan Saiweier Biotechnology Co., Ltd. (Wuhan, Hubei, China).

### 4.12. Data Processing

Data organization was conducted utilizing Microsoft Office software (Microsoft Corporation, Washington, WA, USA). One-way analysis of variance (ANOVA) was performed employing SPSS version 23.0. Each experiment consisted of three biological replicates, and mean comparisons were carried out using Duncan’s multiple range test, with statistical significance denoted by lowercase letters at *p* < 0.05. Graphical representations were generated using Origin 2019b software (OriginLab, Northampton, MA, USA).

## 5. Conclusions

This study identified nine members of *CaAAP* genes in pepper and utilized bioinformatics methodologies to examine gene architecture, evolutionary relationships, and additional related characteristics. Our results demonstrate a pivotal role of *CaAAP6* in response to drought stress. Functional analysis employing VIGS showed that silencing *CaAAP6* led to an elevated accumulation of ROS, accompanied by a reduction in the activity of antioxidant enzymes and the expression of drought-responsive genes. Together, these findings suggest *CaAAP6* is a promising candidate gene for breeding pepper varieties with improved drought tolerance.

## Figures and Tables

**Table 1:** Basic physicochemical properties of *CaAAP* genes.

Gene Name	Gene Symbol	Protein Length	Molecular Mass (KDa)	Instability Index	PI	Predicted Subcellular Localization
*CaAAP1*	*LOC107878850*	492	54.29	29.32	8.45	Plasma membrane
*CaAAP2*	*LOC107878851*	463	51.21	30.23	8.95	Plasma membrane
*CaAAP3*	*LOC107878852*	492	55.08	35.77	9.10	Plasma membrane
*CaAAP4*	*LOC107878853*	468	51.77	31.68	8.77	Plasma membrane
*CaAAP5*	*LOC124885125*	456	50.68	29.42	8.88	Plasma membrane
*CaAAP6*	*LOC107851171*	488	53.83	39.45	8.96	Plasma membrane
*CaAAP7*	*LOC107840162*	478	52.44	27.85	9.00	Plasma membrane
*CaAAP8*	*LOC107867801*	479	52.67	33.96	8.55	Plasma membrane
*CaAAP9*	*LOC107867310*	481	52.76	39.46	8.89	Plasma membrane

**Table 2:** Ka, Ks, and Ka/Ks values and differentiation/replication time of *AAP* gene pairs.

Species	Homologous Gene Pairs	Ka	Ks	Ka/Ks	Divergent/Duplication Time (Mya)
Pepper/Pepper	*CaAAP6*/*CaAAP9*	0.11	0.74	0.15	47.13
Pepper/Arabidopsis	*CaAAP6*/*AtAAP1*	3.13	6.04	0.52	384.71
	*CaAAP6*/*AtAAP8*	0.23	-	-	-
	*CaAAP9*/*AtAAP1*	0.21	2.64	0.08	168.15
	*CaAAP9*/*AtAAP8*	2.61	1.92	1.36	122.29
Pepper/Tomato	*CaAAP9*/*SiAAP1*	0.26	1.58	0.16	100.64
	*CaAAP9*/*SiAAP3*	0.03	0.31	0.10	19.75
	*CaAAP9*/*SiAAP10*	0.13	0.91	0.14	57.96
	*CaAAP6*/*SiAAP3*	0.13	0.77	0.16	49.04
	*CaAAP6*/*SiAAP10*	0.06	0.38	0.15	24.20
	*CaAAP7*/*SiAAP1*	0.08	0.24	0.34	15.29
	*CaAAP5*/*SiAAP6*	2.43	3.82	0.63	243.31

## Data Availability

The original contributions presented in this study are included in the article. Further inquiries can be directed to the corresponding authors.

## Supplementary Materials

The following supporting information can be downloaded at: https://www.mdpi.com/article/10.3390/plants15142167/s1.
