# Supplementary material for: Identification of Pepper AAP Gene Family and Functional Characterization of CaAAP6 in Drought Stress
Source: Plants (Basel). 2026 Jul 14;15(14):2167. doi: 10.3390/plants15142167 (PMC13415076; doi:10.3390/plants15142167)
Supplement: Supplementary file 1 [file plants-15-02167-s001.zip › plants-4412765-supplementary.pdf]

Table S1. Primers used for the VIGS and qRT-PCR in pepper.

Primer name	Forward primers	Reverse primers	Function
CaAAP6	gcTCTAGAagaacaatcaccacaaggact	ggGGTACCatagtttctcttgccggcga	VIGS
CaAAP6	ggactctagactggtacccatggcatcagaatttgagaag	atactagtcagtcgacacccttcatgttcttgaggtttaaag	Subcellular Localization
CaAAP2	gcactcggtaacattgcttt	gctattgaggtcattgacgc	
CaAAP4	cattagaggactgccgagtt	gtgtcctgaacctccagaaa	
CaAAP5	gtgcattgatcttttggcct	aaagcccagtacagacacaa	
CaAAP6	acaagcctaactgggacaaa	gagaatggtggcaaaagcat	
CaAAP7	ctcattttggccactcactg	agggcttgaatttctggaca	
CaAAP8	caagcattcgatgtttccgt	caaccaagttgagctactgc	qRT-PCR
CaAAP9	atagagatgcacattgccca	caagaccctgaatcgatcca	
CaRD22	tgctttcttgcctcgtca	aacacctggttcttcacattct	
CaRD29B	atggaggcacaactgcaccgtc	ggcccaccatgaacttctgcac	
CaDREB2A	gatgcctcggctcctaatca	tgcttgttgcttcacttgct	
Actin	tgtccatctgctctctgttg	caccccaagcacaataagac	

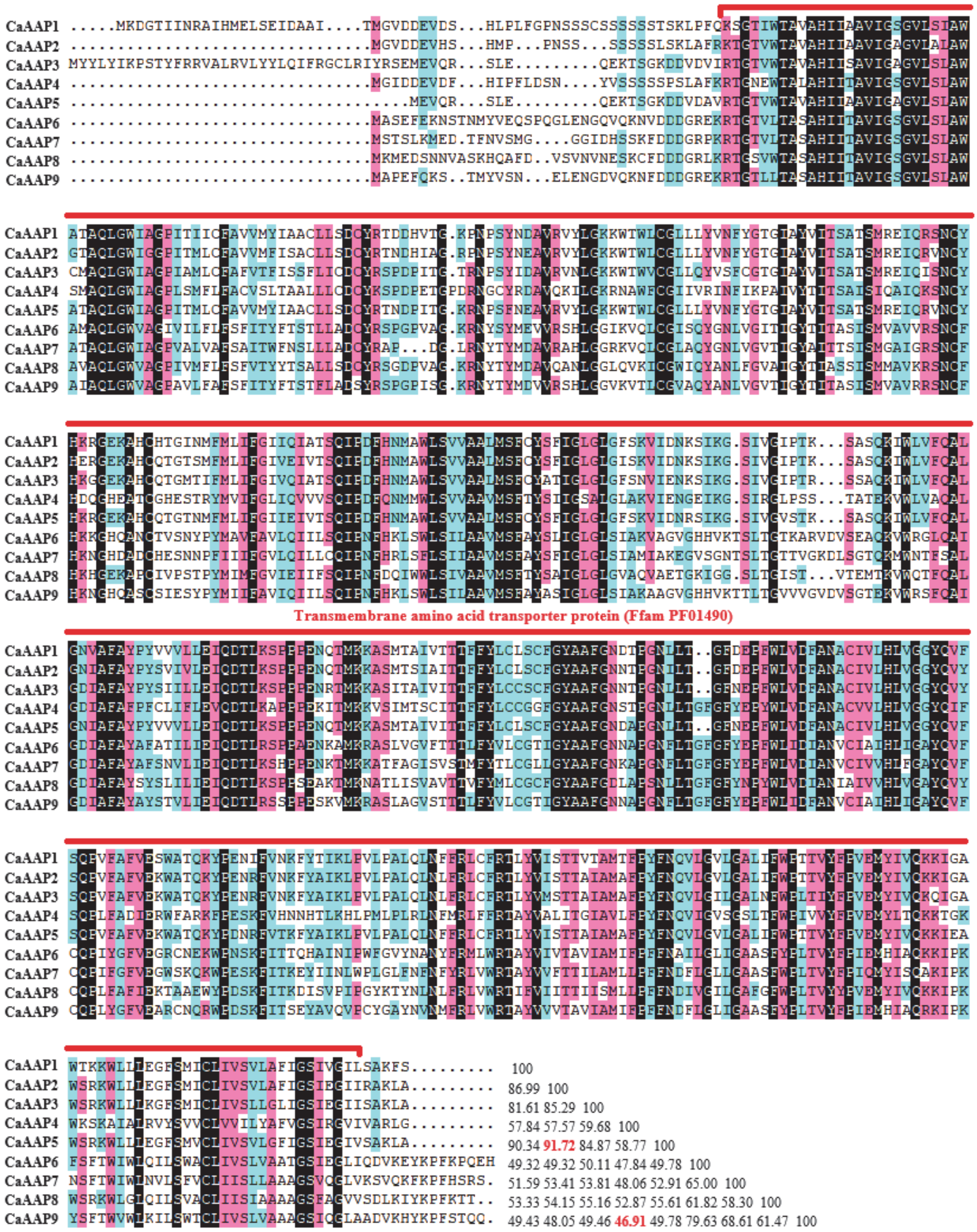

Figure S1. Alignment outcomes of amino acid sequences within the CaAAP family members in pepper. The red line denotes the conservative domain; the numerical values following each sequence indicate the percentage similarity between sequences, with the red numbers highlighting the highest and lowest similarity percentages observed.

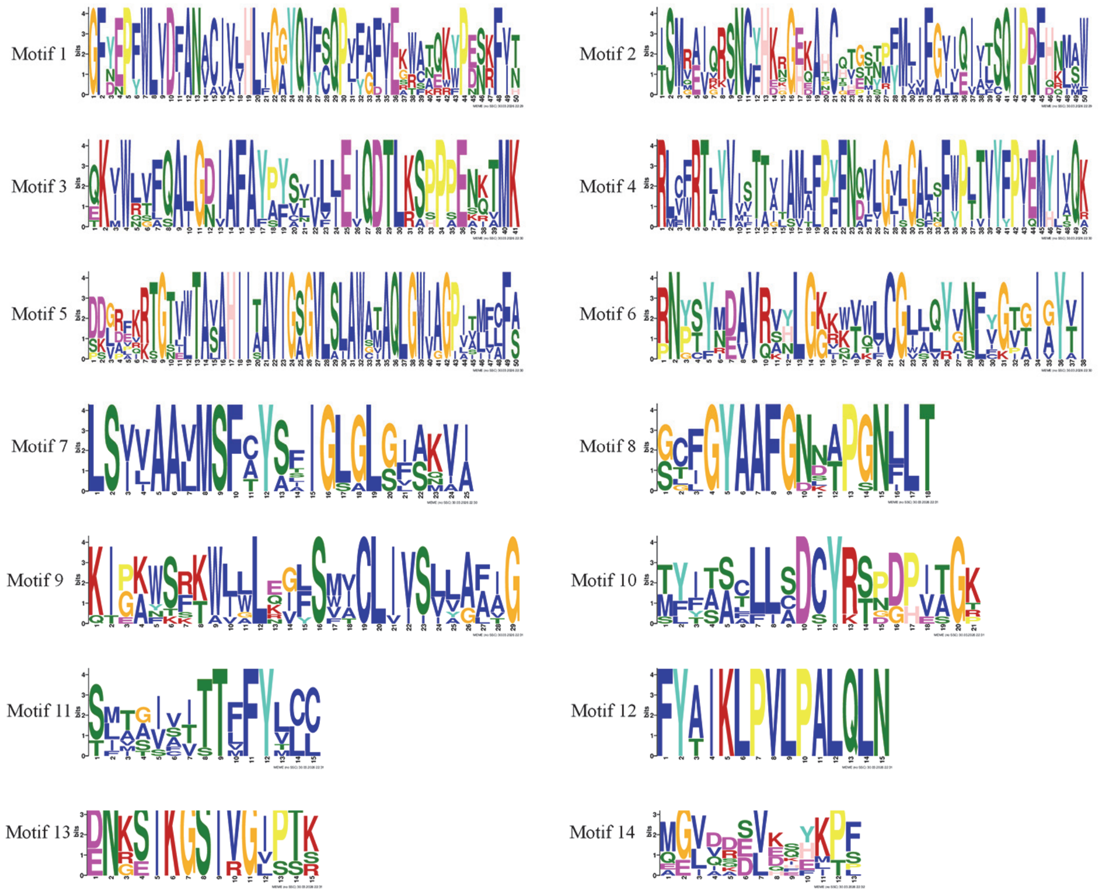

Figure S2. 14 conserved motifs identified within the CaAAP protein sequence. The vertical axis denotes the degree of base conservation, with higher values indicating greater conservation at a given position. The horizontal axis represents the sequence position, organized sequentially from the 5' end to the 3' end.

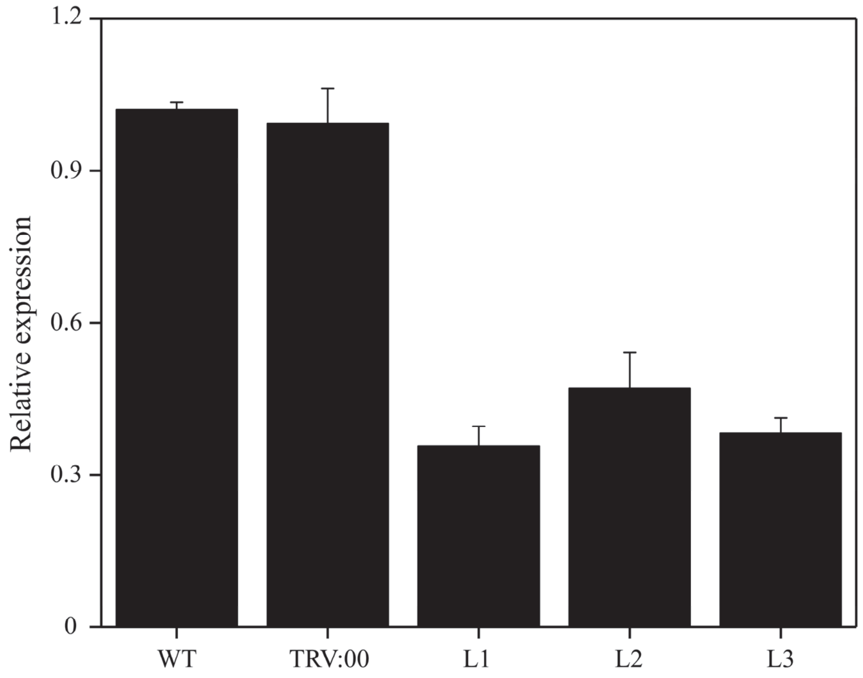

Figure S3. Analysis of plant silencing efficiency in TRV2:*CaAAP6* plants.
